# Revisiting the efficiency and necessity of adenotonsillectomy in children with mild obstructive sleep apnea: a systematic review and meta-analysis

**DOI:** 10.1007/s00405-025-09380-2

**Published:** 2025-04-07

**Authors:** Omar Alomari, Sinem Nur Ertan, Muhammed Edib Mokresh, Elif Nur Arı, Maryam Pourali, Adnan Ali, Seljan Sadigova, Ghazaleh Kokabi Ghahremanpour, Melis Demirag Evman

**Affiliations:** 1 Hamidiye International School of Medicine, University of Health Sciences, Istanbul, 3400 Türkiye; 2 Hamidiye School of Medicine, University of Health Sciences, Istanbul, 3400 Türkiye; 3 Department of Otolaryngology Head and Neck Surgery, Kartal Dr. Lutfi Kirdar Training and Research Hospital, Istanbul, Turkey; 4 Hamidiye International Faculty of Medicine, University of Health Sciences, Istanbul, 34668 Türkiye

**Keywords:** Adenotonsillectomy, Sleep apnea, Meta-analysis, Quality of life, Sleep

## Abstract

**Background:**

Adenotonsillar hypertrophy is the leading cause of obstructive sleep apnea (OSA) in children, with adenotonsillectomy (AT) being the most common surgical treatment. Although AT is widely performed, its efficacy in treating mild OSA remains uncertain. Current literature suggests that children with mild OSA might benefit from non-surgical management, but there is a lack of evidence ND studies evaluating the outcomes of AT specifically for mild OSA. The aim of this systematic review and meta-analysis is to provide conclusive insights into the effectiveness of adenotonsillectomy in improving health outcomes and quality of life for children with mild obstructive sleep apnea.

**Methods:**

PubMed, Scopus, Embase, Cochrane, and Web of Science databases have been searched for relevant studies. We included original studies that evaluated the safety or effectiveness of AT in the management of mild OSA among pediatric patients. For quantitative analysis, data were synthesized using a random-effects model in R (version 4.3.3), and heterogeneity was assessed using statistical methods including the restricted maximum-likelihood estimator and the I2 statistic. We also conducted analyses of change scores and covariance to estimate the effect of AT on the severity of mild OSAS.

**Results:**

Our review included 27 studies after screening 1851 citations. The meta-analysis demonstrated significant improvements with AT for mild OSA. The Pediatric Sleep Questionnaire scores improved with a mean difference (MD) of -0.32 (95% CI [-0.39; -0.25], *p* < 0.001). AHI decreased significantly with an MD of -1.45 (95% CI [-2.11; -0.80], *p* < 0.001). Comparison with watchful waiting revealed AT to be more effective: AHI showed an MD of -1.22 (95% CI [-1.92; -0.53], *p* < 0.001), and the arousal index had an MD of -1.73 (95% CI [-2.95; -0.51], *p* = 0.005). Safety data indicated that while AT is generally safe, it is associated with minor complications such as postoperative desaturation and occasional bleeding. Long-term serious adverse events were rare.

**Conclusion:**

AT effectively improves symptoms in children with mild OSA, outperforming watchful waiting in several key metrics. This review supports AT as a viable option but underscores the importance of considering individual patient factors in treatment decisions.

## Introduction

Obstructive sleep apnea syndrome (OSAS) entails intermittent obstruction of the upper airway during sleep, resulting in episodes of hypoxia, hypercapnia, heightened respiratory effort, notable swings in intrathoracic pressure, and frequent arousals that fragment sleep [1]. Pediatric OSA is a prevalent condition in childhood, impacting approximately 1–5% of children [2].

The diagnosis of pediatric OSA relies on assessing the number of apnea and hypopnea events per hour, referred to as the apnea-hypopnea index (AHI), through polysomnography (PSG). In most studies, an AHI value of 1 or greater is considered abnormal. OSA is part of the spectrum of sleep disorders known as obstructive sleep-disordered breathing (oSDB), encompassing a range of severity from primary snoring to severe OSA [3].

Abnormal findings in polysomnography are categorized into specific groups to facilitate targeted treatment approaches. Mild OSAS is characterized by an AHI of 1–2, SaO2 nadir of 90–94%, or highest PETCO2 of 46–49 mmHg. Moderate OSAS presents with an AHI of 3–5, SaO2 nadir of 85–89%, or highest PETCO2 of 50–54 mmHg. Severe OSAS is defined by an AHI greater than 5, SaO2 nadir below 85%, and highest PETCO2 exceeding 54 mmHg. These distinctions enable healthcare providers to implement appropriate treatments, ranging from lifestyle modifications to continuous positive airway pressure (CPAP) therapy or surgical interventions, depending on the severity of the condition [4].

The predominant cause of OSA in children is adenotonsillar hypertrophy, and the primary surgical intervention for this sleep disorder is adenotonsillectomy (AT). Adenotonsillar hypertrophy stands out as the most frequently identified risk factor for childhood obstructive sleep apnea syndrome. Notably, AT ranks among the most prevalent surgical procedures performed in children worldwide [5].

If OSAS is left untreated, it can significantly impact children’s quality of life [6].Research has linked oSDB with behavioral and neurocognitive challenges [7]. Cognitive assessments, whether symptom-based or relying on PSG, have indicated lower intelligence test scores among children with oSDB compared to those without [8]. Furthermore, untreated OSAS, the most severe form of oSDB, poses risks of significant health complications, including failure to thrive and cardiovascular diseases such as hypertension, cor pulmonale, and left ventricular hypertrophy [9].

Contrary to a uniform approach, certain reports and studies suggest that AT may not always be the initial treatment choice for otherwise healthy children with mild OSA [10–12]. Both research findings and clinical observations indicate that children experiencing less severe forms of OSA may show improvement without resorting to surgical intervention. However, it is noteworthy that there is a lack of controlled studies evaluating the potential benefits and risks associated with adenotonsillectomy in managing obstructive sleep apnea syndrome. Beyond considerations of treatment efficacy, there are also perioperative and postoperative risks to take into account, including issues such as pain, hemorrhage, and respiratory compromise [6].

The objective of our systematic review and meta-analysis is to provide conclusive insights into the efficacy of adenotonsillectomy for children with mild obstructive sleep apnea, where a consensus is currently lacking. Our aim is to discern the extent of benefit that adenotonsillectomy offers in terms of improving the health and overall quality of life for affected children. Through a comprehensive analysis of existing research and data, we strive to contribute to a better-informed decision-making process regarding the optimal management of mild obstructive sleep apnea in children.

## Methods

We used a systematic review strategy to perform complete literature retrieval across several academic databases to explore scientific papers relevant to the outcomes of ATE among children with mild obstructive sleep apnea. This review followed the Preferred Reporting Items for Systematic Reviews and Meta-Analysis (PRISMA) 2020 standards, guaranteeing a robust and uniform methodology [13]. We submitted the research protocol for this systematic review to the International Prospective Registry of Systematic Reviews (PROSPERO) database (www.crd.york.ac.uk/prospero/*)* and assigned the PROSPERO ID: **CRD42024512651**. All working group members agreed on the study protocol before beginning the literature search.

### Search strategy and study selection

We conducted a systematic search by title and abstract on the following databases: Medline (through PubMed), Scopus, Web of Science, and Embase up until the 25th of September 2024. We used the MeSH database to retrieve the synonyms of our search strategy, and the terms were combined using “OR” and “AND” boolean operators, in accordance with the Cochrane Handbook for Systematic Reviews (Chap. 4.4.4) [14]. The terms were as follows: “mild sleep-disordered breathing” OR “mild sleep apnea” OR “sleep-disordered breathing”) AND TITLE-ABS-KEY(“tonsillectomy” OR “adenoidectomy” OR “adenotonsillectomy”) AND TITLE-ABS-KEY(“children” OR “pediatric population”). Following the removal of duplicates, a team of four authors screened the retrieved studies based on our predefined eligibility criteria using titles and abstracts. Subsequently, the list of included studies was subjected to further scrutiny by two authors. Studies deemed relevant and any conflicts were subjected to full-text screening.

### Eligibility criteria

We incorporated studies that assessed the efficiency of ATE in the management of mild obstructive sleep apnea. There were no restrictions placed on language or the year of publication. To ensure the quality and relevance of the included studies, we excluded duplicate publications, reviews, letters to editors, book chapters, animal and laboratory studies, and in vitro investigations.

### Data extraction

Six authors undertook the task of extracting data from the included studies and entering the collected information into a pre-piloted excel spreadsheet. To guarantee data accuracy and consistency, a seventh author meticulously reviewed the completed extraction sheet, reconciled any discrepancies, and validated the precision of the data. The data extraction encompassed several key elements, starting with study characteristics such as the first author’s name, publication year, study design, and geographical location. Population characteristics, including sample size, gender distribution, age demographics. Several apnea related parameters such as AHI index, OSA-18 Quality of Life Survey, Paediatric Sleep questionnaire (PSQ), and oximetry results have also been extracted.

For the quantitative analysis, the same authors independently extracted information; AHI, OHAI, OSA-18, O2 nadir, arousal index, PSQ, mean duration of follow-up, When required Data reported using different formats were converted into mean and standard deviation values using the website developed by McGrath et al. which is an online tool used to facilitate the estimation of sample mean and standard deviation, ensuring consistency in the data presentation and analysis [15].

### Critical appraisal tool and risk of bias assessment

Two authors (MEM and OA) conducted an assessment of the risk of bias in the eligible included studies, comprising 19 Observational studies and 7 relevant RCTs. The National Institutes of Health (NIH) Quality Assessment Tool for Observational Cohort and Cross-sectional Studies was employed for this purpose [16]. The methodological quality of the included clinical trial was assessed by using the Cochrane Collaboration’s tool for assessing the risk of bias in randomized trials RoB2 tool [17]. The use of the RoB2 tool allowed for a systematic examination of various domains that could introduce bias into the studies. These domains include the randomization process, deviations from intended interventions, missing outcome data, measurement of the outcome, and selection of the reported result. Each domain was scrutinized to determine the potential risk of bias, ensuring a comprehensive evaluation of the included studies. Any discrepancies in their assessments were resolved through discussions among researchers until a consensus was reached.

### Data analysis

Meta-analysis was conducted using R (version 4.3.3) with the statistical packages “tidyverse” and “meta” [18]. Continuous variables were summarized using mean values with standard deviations, while dichotomous variables were presented as frequencies with percentages. A random-effects model was utilized to amalgamate the data. Heterogeneity was assessed using various statistical methods, including the restricted maximum-likelihood estimator (τ2) [19] the I2 statistic [20], and the Q-test [21]. Analysis of final (follow-up) scores, analysis of change scores calculated by subtracting the follow-up from the baseline score and analysis of covariance (ANCOVA) recovered effect estimates model, which adjusts for the baseline scores have been reported [22, 23].

$$\:{SD}_{Change}\:=\:\sqrt{{\left({SD}_{B\:}\right)}^{2}\:+\:{\left({SD}_{F}\right)}^{2}\:-\:2\:\times\:\:r\:\times\:\:{SD}_{B}\:\times\:\:{SD}_{F}}$$

While the within-group correlation was calculated by the following formula:

$$\:r\:=\:\frac{{SD}_{B}^{2}\:+\:{SD}_{F}^{2}\:-\:{SD}_{Change}^{2}}{2\:\times\:\:{SD}_{B}^{2}\:\times\:\:{SD}_{F}^{2}}$$ [24].

## Results

**Fig. 1:**
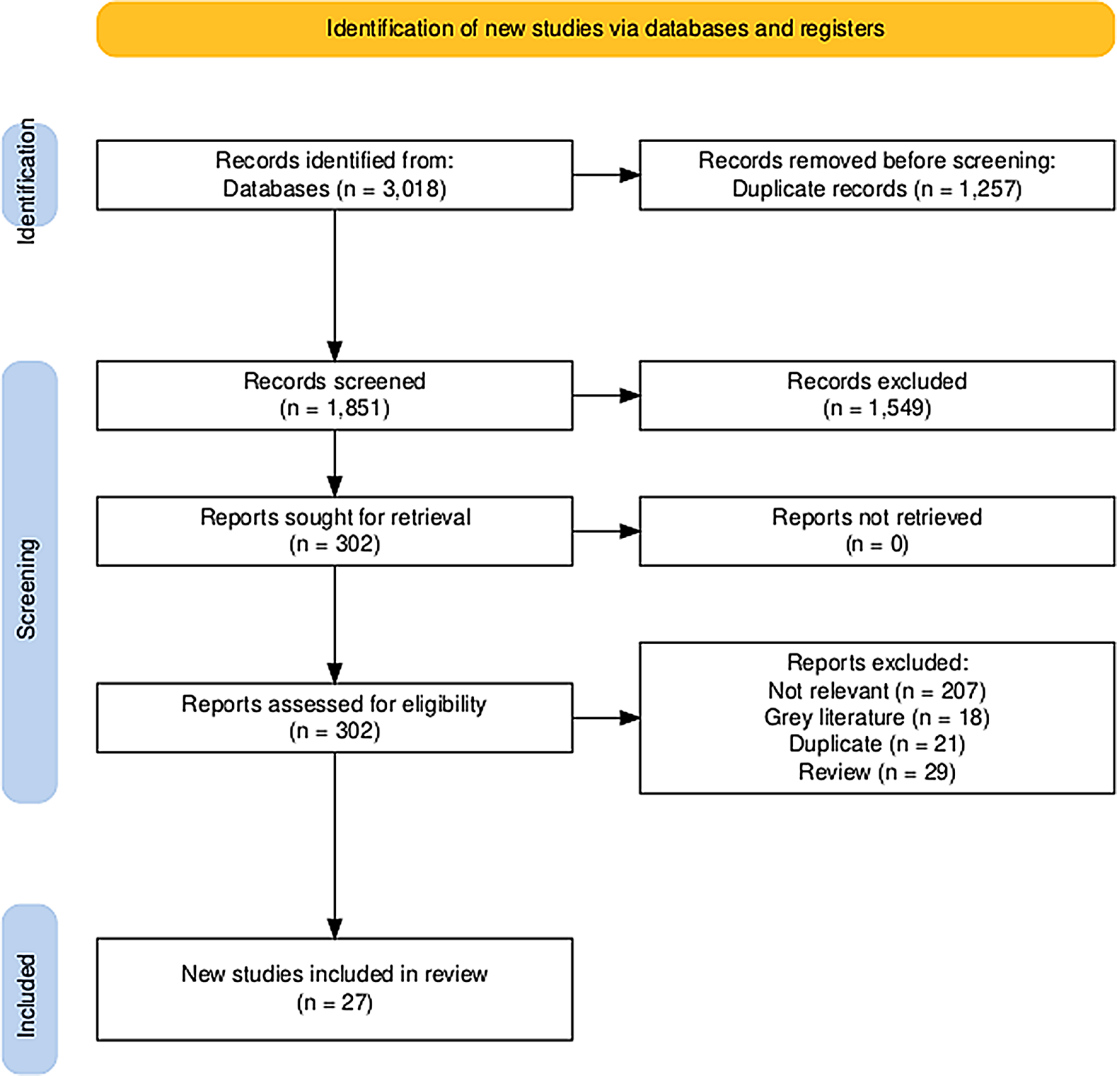
PRISMA flow diagram illustrating the study selection process. The diagram outlines the number of records identified, screened, assessed for eligibility, and included in the final analysis, along with reasons for exclusion at each stage

Our initial search yielded 1851 unique citations, out of which 302 full-text articles were assessed for eligibility. Eventually, 275 articles were excluded, and we included 27 unique studies in our analysis [25–51]. The detailed search process and the selection of studies are elaborated in the accompanying PRISMA diagram (Fig. 1). The included studies encompass a diverse range of designs, including prospective observational studies, randomized controlled trials, cross sectional studies and retrospective cohort studies across various countries, predominantly in the USA, but also including Canada, England, Australia, Hong Kong, China, Taiwan, and India. The sample sizes vary widely, from small cohorts of around a dozen (*n* = 9) to larger groups of over 600 participants. The age of patients ranges from preschool-aged children to those up to 17 years old, with most studies focusing on young children. Gender distributions are generally balanced, though some studies have a higher percentage of males. Racial and ethnic compositions vary, reflecting the geographic diversity of the studies, with notable proportions of Hispanic/Latino, Black, White, and other racial/ethnic groups. The detailed baseline characteristics of the included studies and patients are presented on Table 1.

**Table 1:** Baseline characteristics of the included studies

Author	Year	Country	Study Design	Sample Size	Age (years)	Sex	BMI	Aim	Main Outcome
Owens et al.	2000	USA	Prospective Observational	Mild OSAS: 9, Moderate OSAS: 9	G1 Mean Age = 8.4 ± 2., G2 Mean Age = 6.1 ± 0.84	(12 males, 6 females)	NE	To examine a group of children with documented OSAS, utilizing a comprehensive neuropsychological battery and assess changes post-adenotonsillectomy.	Results suggest mild deficits in neuropsychological measures of executive functioning/attention and motor skills in children with OSAS improved post-T&A.
Mitchell et al.	2005	USA	Prospective Observational	OSAS: 43, Mild SDB:18	Mean age (Range) 7 (3–17) / 7 (3–15)	Male 69% / 67%	BMI 23 (12–48) / 21 (12–43)	To evaluate the relationship between quality of life and the relative severity of SDB and compare changes in quality of life post-T&A.	Both groups of children showed a dramatic improvement in quality of life post-T&A.
Nixon et al.	2005	Canada	Prospective Observational	Mild OSA: 5, Severe OSA: 5	Age 4.9 (3.2–5.8) / 3.9 (2.6–5.1)	Females (40%)	Weight percentile 83 (6–88) / 10 (1–88), Height percentile 75 (13–87) / 25 (3–35)	To compare the nature and severity of SDB in children with mild and severe OSA on the first night following adenotonsillectomy.	Despite removal of obstructing lymphoid tissue, upper airway obstruction occurred on the first postoperative night in children with OSA.
Chervin et al.	2005	USA	Prospective Observational	AT: 78, Control:27	8.1 ± 1.8 / 9.3 ± 2.0	Male: 41/19	20.0 ± 5.1 / 18.9 ± 2.9	To study children with SDB before and after clinically indicated adenotonsillectomy or unrelated surgical care.	Neurobehavioral morbidity improved by 1 year post-surgery, but lack of correspondence between SDB measures and neurobehavioral outcomes.
Hogan et al.	2007	England	Observational Longitudinal Cohort	Control: 14, SDB: 19	Month 58 ± 14 / 61 ± 14	Male/Female: Control 8/6, SDB 9/10	Preoperative BMI z score, mean ± SD Control = 0.28 ± 1.1 / SDB = -0.16 ± 1.6	To determine whether amelioration of SDB through T&A would reduce middle cerebral artery velocity with improvements in cognition and behavior.	Significant improvement in PSQ score post-operatively and significant increase in mean overnight oxyhemoglobin saturation.
Mitchell	2007	USA	Prospective Observational	79	6.3 (range 3.0–15.8)	Male: 40	9% underweight, 81% normal weight, 10% at risk of being overweight	To evaluate the outcome of T&A for OSA in children using polysomnography data supplemented by OSA-18 quality of life instrument.	T&A results in significant improvement in respiratory parameters and quality of life in children with OSA.
Mitchell et al.	2007	USA	Prospective Observational	Mild SDB = 17, OSA = 23	Mild SDB 7.3 (3.2–12.9), OSA 6.9 (3.1–14.9)	Male: 62% / 48%	BMI 22 (12–44) / 23 (12–46)	To compare changes in behavior post-T&A in children with either mild SDB or OSA.	Behavioral problems are prevalent in children with either mild SDB or OSA, with significant improvements in behavior post-T&A.
Powell et al.	2011	UK	Prospective Observational	OSA = 22	Mean age: 61 months	15 boys and 7 girls	NE	To assess the quality of life in UK children with SDB undergoing T&A using the OSA 18 questionnaire.	Significant improvements in quality of life post-T&A, especially in sleep disturbance and caregiver concern.
Meyer et al.	2012	USA	Retrospective Observational	13	6 months to 11 years, median age at T&A of 3 years	6 girls (46%) and 7 boys	Mean BMI = 17.93	To investigate the effects of T&A on pediatric patients with PWS who have SDB, specifically OSA or obstructive hypoventilation.	Significant improvement in breathing in patients with mild to moderate OSA or obstructive hypoventilation post-T&A. Some severe OSA cases did not improve.
Lee et al.	2014	China	Prospective Observational	144	Mean age 7.0 ± 3.6	109 male, 35 female	Subgroup analysis done	To explore QoL improvement post-T&A in children with SDB, specifically OSA, and analyze impact of factors like gender, age, adiposity, and disease severity on QoL.	Significant improvement in QoL post-T&A measured by OSA-18 questionnaire in children with SDB.
Baldassari	2013	USA	Prospective Observational	AT group = 30, Observation group = 34	80.8 ± 33.7 / 80.0 ± 41.9 months	Male: Female, 16:14 / 20:14	18.9 ± 6.1 / 20.2 ± 6.2	To determine the impact of T&A vs. observation on QoL in children with mild OS.	Significant improvement in QoL in children with moderate OSA-18 score post-T&A, indicating they are good candidates for T&A.
Nixon et al.	2015	Australia	Prospective Observational	Control: 28 children with SDB; Treated: 23, Untreated: 22	4.30 ± 0.20 / 4.10 ± 0.20 / 4.40 ± 0.20	44%, 78%, 55%	16.40 ± 0.30 / 16.50 ± 0.50 / 17.20 ± 0.40	To compare sleep and respiratory parameters in preschool children to examine the effects of treatment or no treatment	The long-term results highlight the need for greater caution regarding the persistence or possibility of recurrence of SDB
Gan	2015	United Kingdom	Cross-Sectional	51	Mean age 4 (2–13)	NE	NE	To investigate whether children with mild/moderate SRBD have respiratory problems during the first post-op night	Same-day discharge for otherwise-healthy children over 4 years old having AT for mild/moderate SRDB appears to be safe
Trosman et al.	2016	United States	Retrospective Observational	AT = 18, Watchful Waiting = 44	3.1 (2.3) / 4.5 (2.7)	Male = 11:7 / 26:18	NE	To compare outcomes following AT vs. observation in children with mild OSA based on polysomnography results	The mean apnea hypopnea index after AT improved from 3.5 to 2.69, while the observation group’s index worsened from 3.09 to 5.18, but this difference was mostly seen in non-obese and non-syndromic children
Chawla et al.	2021	Australia	Prospective Observational	Obstructive sleep apnea group = 52, Primary snoring group = 39	3.8 (± 0.73) / 4.01 (± 0.65)	Male = 28:24 / 24:15	NE	To evaluate the difference in neurocognitive and behavioral parameters in symptomatic preschool children between PSG-diagnosed OSA and PS	No significant differences were found in neurocognitive or behavioral parameters for children with OSA versus those with PS
Kang et al.	2017	Taiwan	Retrospective Observational	610 children (AHI < 1, *n* = 82; 1 < AHI < 5, *n* = 228; 5 < AHI < 10, *n* = 97; AHI > 10, *n* = 203)	7.0 ± 3.0 / 7.1 ± 3.0 / 7.0 ± 3.5 / 7.6 ± 3.7	Male 71.5% (436/610)	24.8% (151/610) Obese	To find the effects of disease severity on postoperative complications of AT for children under 18 with SDB	Children with more severe OSA are at an increased risk of perioperative respiratory complications. Pediatric OSA does not appear to be correlated with postoperative bleeding or major respiratory complications
Wang et al.	2017	China	Randomized Clinical Trial	Given montelukast for 12 weeks after AT = 29, No treatment after AT = 29	7.93 ± 2.24 / 7.41 ± 2.18	Male = 16:13 / 14:15	NE	To evaluate the treatment effect of montelukast in children with OSA after AT	NE
Mir et al.	2019	India	Randomized control study	AHI < 5 (*n* = 15), AHI ≥ 5 (*n* = 18)	9 ± 2.97	78.8% males	NE	To assess the prevalence of neurocognitive and behavioral dysfunction in Indian children with SDB and to evaluate the effect of AT	Patients with a baseline AHI > 5/h and those who had complete resolution of SDB (postoperative AHI < 1/h) showed improvement in more subscales than patients with baseline AHI < 5/h and patients with incomplete resolution of SDB
Waters et al.	2020	UK	Randomized controlled study	AT vs. No AT	Months 46.5 ± 8.8 / 47.8 ± 8.8	Male = 52:47 / 57:34	NE	To evaluate outcomes 12 months after adenotonsillectomy compared with no surgery in preschool children symptomatic for obstructive sleep apnea	Improvements were seen after adenotonsillectomy in sleep and behavior using polysomnogram monitoring and parental questionnaires
Au et al.	2021	Hong Kong	Randomized controlled study	Early surgical intervention (*n* = 35), Watchful waiting (*n* = 36)	Baseline + change from baseline 8.4 ± 1.6 & 0.9 ± 0.2 / 8.4 ± 1.4 & 0.8 ± 0.3	Male (%) 54.3 / 69.4	NE	To evaluate inattention and behavioral outcomes following surgery versus watchful waiting (WW) in school-aged children with mild obstructive sleep apnoea (OSA)	Despite improvements in PSG parameters and parent-reported symptoms, surgical treatment did not lead to parallel improvements in objective attention measures in school-aged children with mild OSA
Redline et al.	2023	USA	Randomized controlled study	AHI < 1 *n* = 311, AHI > 1 *n* = 148	6.0 (4.0–7.0) / 6.0 (4.0–8.0)	Female 150/80	NE	To assess whether a combination of clinical characteristics differentiates children with primary snoring from children with mild OSA	Primary snoring and mild OSA were difficult to distinguish without polysomnography. Mild OSA vs. snoring alone did not identify a clinical group of children who may benefit from AT for obstructive sleep-disordered breathing
Fehrm et al.	2020	Sweden	Randomized controlled study	Early AT *n* = 29, Watchful waiting *n* = 31	Mean (SD) months 39 (8) / 37 (11)	Male *n* = 15 / 19	NE	To determine whether ATE is more effective than watchful waiting for treating otherwise healthy children with mild to moderate OSA	This trial found only small differences between groups regarding changes in OAHI, but large improvements in quality of life after ATE. Children with moderate OSA should be considered for ATE
Huang et al.	2006	Taiwan	Nonrandomized controlled study	Surgical tx = 25, MPH tx = 27	8.08 (1.28) / 8.19 (1.73) / 8.07 (2.30) / 8.85 (2.13)	Male *n* = 23/24/12/16	NE	To evaluate if treating OSA has similar results as MPH, a commonly used treatment for ADHD	Recognition and surgical treatment of underlying mild SDB in children with ADHD may prevent unnecessary long-term MPH usage
Waters et al.	2019	Australia	Randomized control trial	Early AT *n* = 55, Routine surgery *n* = 52	NE	NE	NE	To assess if early AT would show changes in neurocognitive function in children with OSA	This study will provide long-term follow-ups on whether kids with OSA benefit from early AT depending on the timing of the surgery
Kevat et al.	2023	Australia	Nonrandomized control trial	Early Surgery (*n* = 68), Routine Surgery (*n* = 58)	Mean, SD: 49.46 (± 8.44) / 48.97 (± 8.23)	Female 28 (45.9%) / 24 (47.06%)	BMI-for-age Z-score (median, IQR): 0.22 (0.43 to 0.97) / 0.56 (0.24 to 1.21)	To assess the impact of adenotonsillectomy on growth trajectories in preschool-aged children with mild–moderate obstructive sleep apnea using longitudinal data from a multicenter randomized controlled trial	Adenotonsillectomy leads to a notable but time-limited increase in BMI and weight in preschool children with mild–moderate obstructive sleep apnea in the months immediately following the surgery
Redline et al.	2023	US	Randomized clinical trial	Early adenotonsillectomy (*n* = 231), Watchful waiting (*n* = 227)	Age, median (IQR), y: 6 (4–8) / 6 (4–8)	Male: 112 (48.5%) / 112 (49.3%)	BMI, mean (SD): 17.2 (2.6) / 17.0 (2.5)	To determine the impact of early adenotonsillectomy on health-related quality of life in children with obstructive sleep apnea compared to a watchful waiting approach	Early adenotonsillectomy significantly improved the quality of life in children with obstructive sleep apnea compared to the watchful waiting approach, especially in sleep-related and behavioral domains

(Adenotonsillectomy), AT (Adenotonsillectomy), BMI (Body Mass Index), MPH (Methylphenidate), OSA (Obstructive Sleep Apnea), OSAS (Obstructive Sleep Apnea Syndrome), PSQ (Pediatric Sleep Questionnaire), PWS (Prader-Willi Syndrome), SDB (Sleep-Disordered Breathing), SRBD (Sleep-Related Breathing Disorder), T&A (Tonsillectomy and Adenoidectomy), and WW (Watchful Waiting)

### Efficacy of adenotonsillectomy

**Fig. 2:**
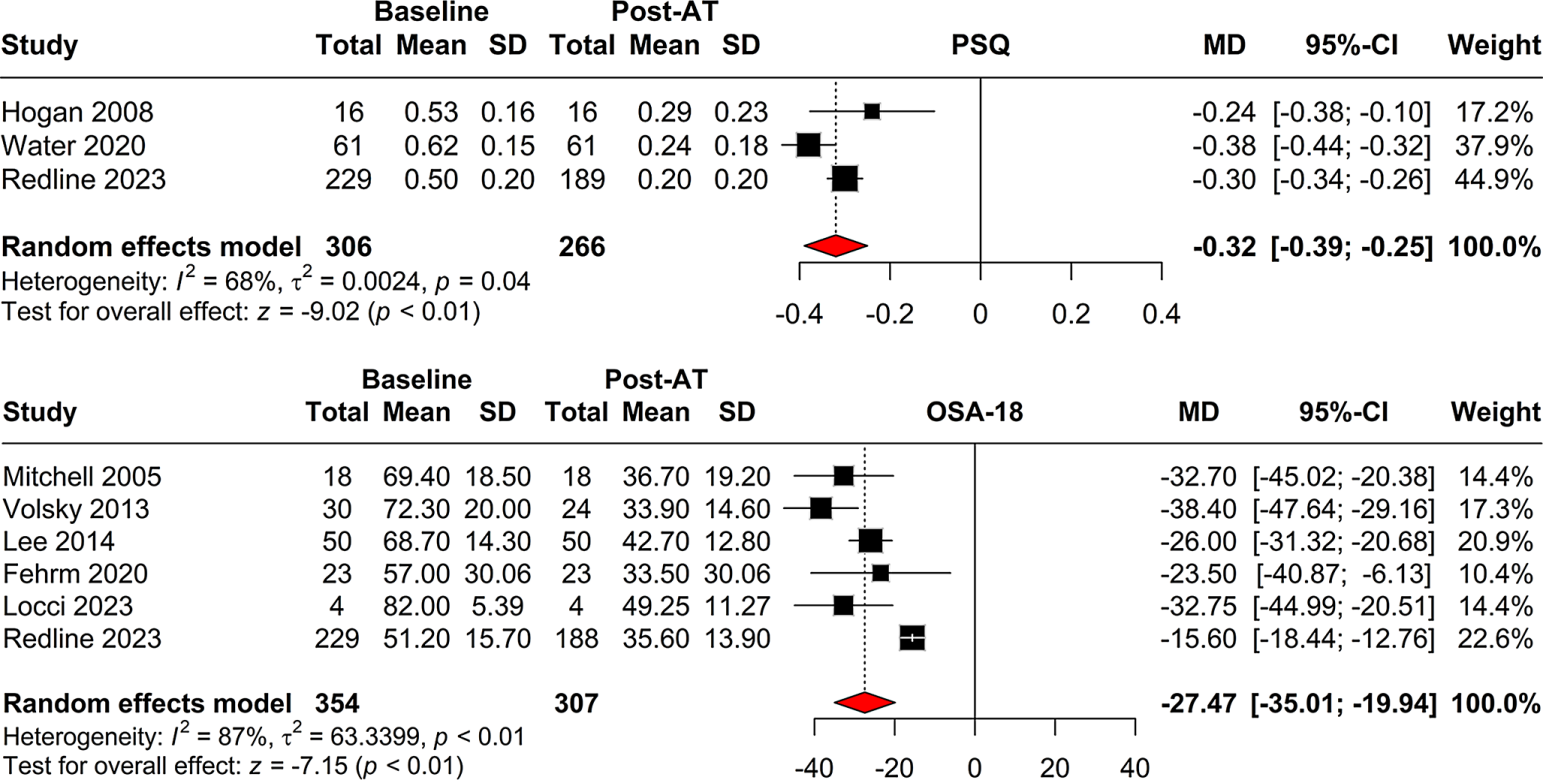
Meta-analysis of the efficacy of adenotonsillectomy in improving Pediatric Sleep Questionnaire (PSQ) and OSA-18 Quality of Life Survey scores

The pooled analysis for the PSQ included three studies with a random effects model of change from baseline to follow-up indicating a mean difference (MD) of -0.32 with a 95% confidence interval (CI) of [-0.39; -0.25], which was statistically significant (*p* < 0.001) (Fig. 2). Heterogeneity was moderate (I^2 = 68.5%). The meta-analysis for OSA-18 Quality of Life Survey included six studies. The random effects model showed a significant change from baseline with MD of -27.47 (95% CI = [-35.01; -19.94], *p* < 0.001). However, the heterogeneity was high (I^2 = 86.7%) (Fig. 2).

**Fig. 3:**
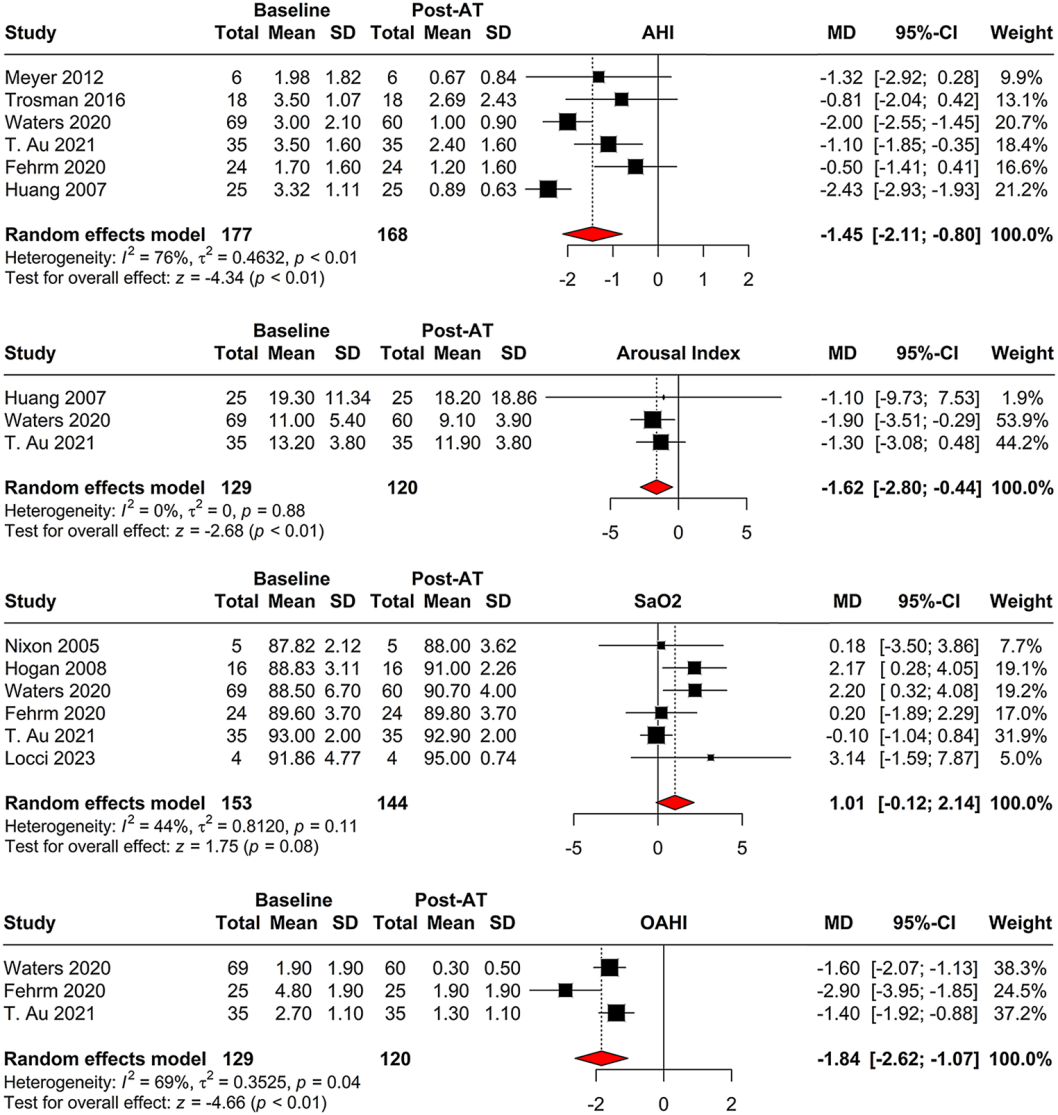
Meta-analysis of polysomnographic outcomes following adenotonsillectomy. The forest plots display changes in Apnea-Hypopnea Index (AHI), arousal index, oxygen saturation (SaO2), and Obstructive Apnea-Hypopnea Index (OAHI) from baseline to post-surgery

The pooled analysis of AHI change from baseline to post- AT involved six studies. The analysis showed an MD of -1.45 (95% CI = [-2.11; -0.80], *p* < 0.001) with substantial heterogeneity (I^2 = 75.8%) (Fig. 3). Arousal index changes were evaluated in three studies, yielding an MD of -1.62 (95% CI = [-2.80; -0.44], *p* = 0.007) with no heterogeneity observed (I^2 = 0.0%) (Fig. 3). For oxygen saturation (SaO2), six studies showed an MD of 1.01 (95% CI = [-0.12; 2.14], *p* = 0.080) with moderate heterogeneity (I^2 = 43.7%) (Fig. 3). The pooled analysis for obstructive apnea-hypopnea index (OAHI) included three studies. The MD was − 1.84 (95% CI = [-2.62; -1.07], *p* < 0.001) with moderate heterogeneity (I^2 = 68.5%) (Fig. 3).

### Adenotonsillectomy vs. Watchful waiting

**Fig. 4:**
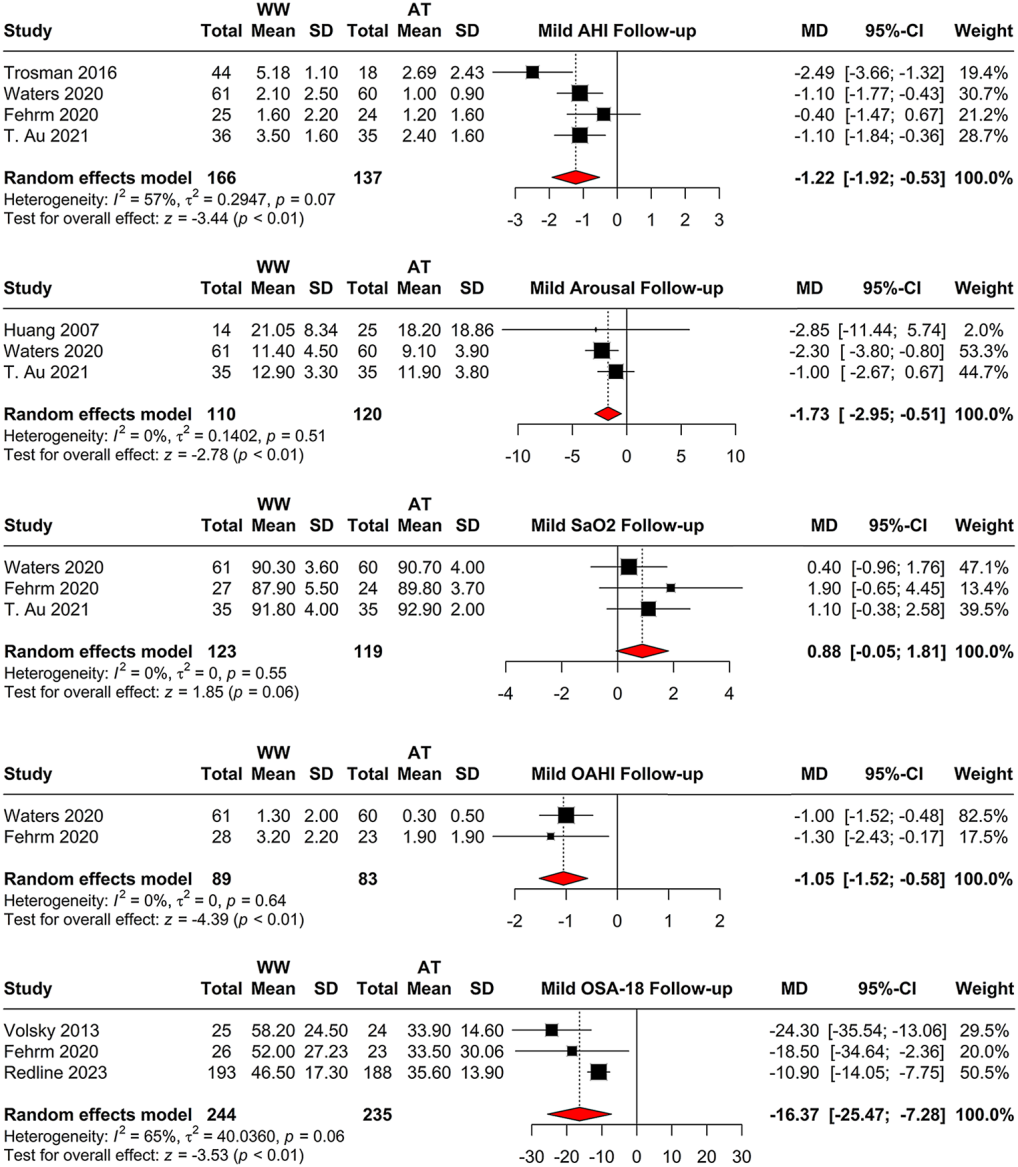
Comparison of adenotonsillectomy versus watchful waiting at follow-up. The forest plots depict mean differences in AHI, arousal index, SaO2, OAHI, and OSA scores between the two groups

Comparing adenotonsillectomy with watchful waiting after the follow-up, four studies reported an MD of -1.22 (95% CI = [-1.92; -0.53], *p* < 0.001) for AHI follow-up values, with moderate heterogeneity (I^2 = 56.8%) (Fig. 4). For the arousal index, three studies showed an MD of -1.73 (95% CI = [-2.95; -0.51], *p* = 0.005), with no heterogeneity (I^2 = 0.0%) (Fig. 4). SaO2 follow-up values in three studies resulted in an MD of 0.88 (95% CI = [-0.05; 1.81], *p* = 0.065) with low heterogeneity (I^2 = 0.0%) (Fig. 4). For OAHI, two studies showed an MD of -1.05 (95% CI = [-1.52; -0.58], *p* < 0.001), with minimal heterogeneity (I^2 = 0.0%)(Fig. 4). OSA follow-up values from three studies indicated an MD of -16.37 (95% CI = [-25.47; -7.28], *p* < 0.001), with moderate heterogeneity (I^2 = 64.8%) (Fig. 4).

**Fig. 5:**
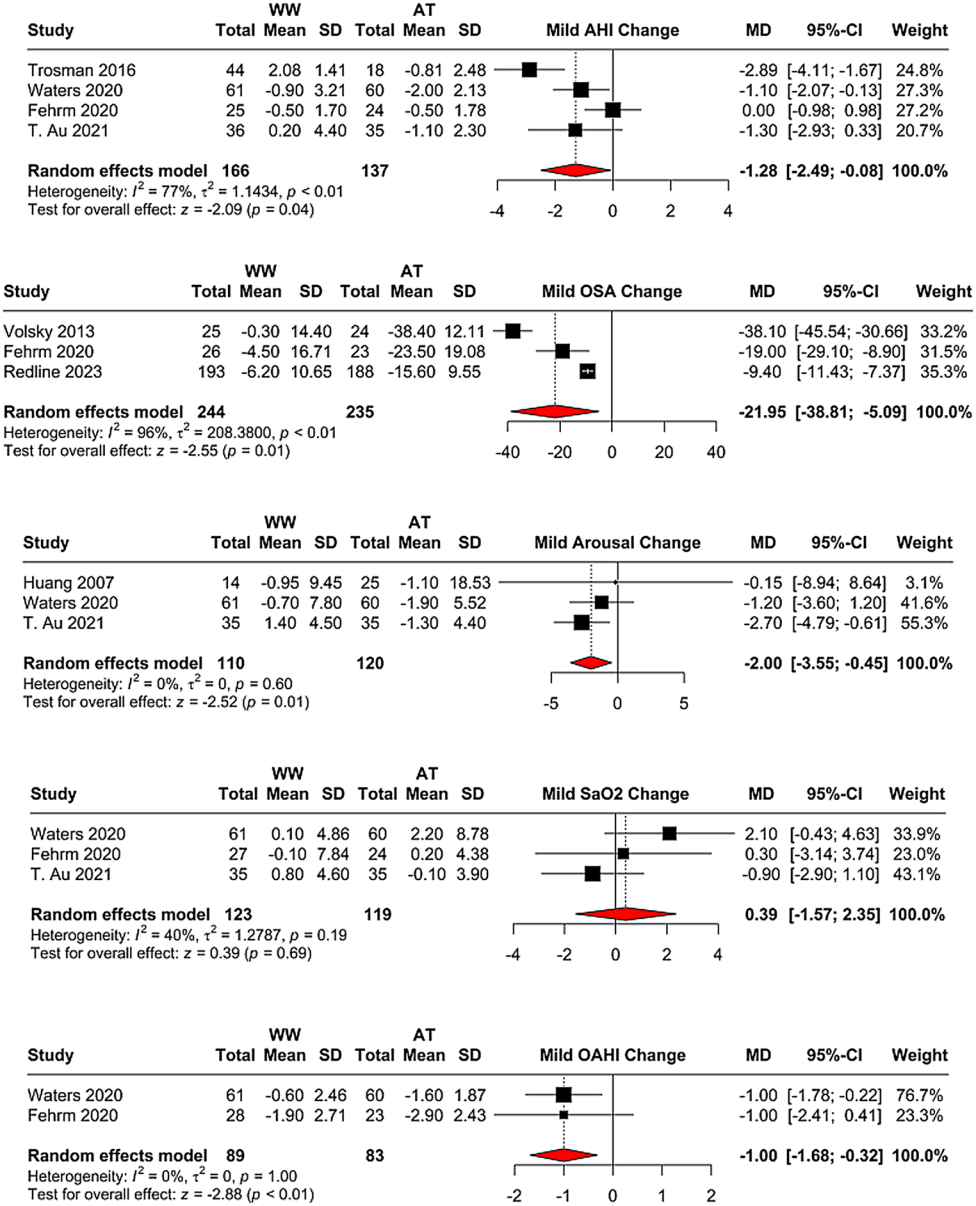
Meta-analysis of change values from baseline in adenotonsillectomy versus watchful waiting. The forest plots present MDs in AHI, arousal index, SaO2, OAHI, and OSA scores

Evaluating change values from baseline between adenotonsillectomy and watchful waiting, four studies indicated an MD of -1.29 (95% CI = [-2.49; -0.08], *p* = 0.037) for AHI, with high heterogeneity (I^2 = 77.3%) (Fig. 5). The arousal index change values from three studies showed an MD of -2.00 (95% CI = [-3.55; -0.45], *p* = 0.012), with no heterogeneity (I^2 = 0.0%) (Fig. 5). SaO2 change values from three studies yielded an MD of 0.39 (95% CI = [-1.57; 2.35], *p* = 0.69), with low heterogeneity (I^2 = 39.8%) (Fig. 5). For OAHI change values, two studies showed an MD of -1.00 (95% CI = [-1.68; -0.32], *p* = 0.004), with no heterogeneity (I^2 = 0.0%) (Fig. 5). OSA change values from three studies indicated an MD of -21.95 (95% CI = [-38.81; -5.09], *p* = 0.011), with high heterogeneity (I^2 = 96.4%) (Fig. 5).

**Fig. 6:**
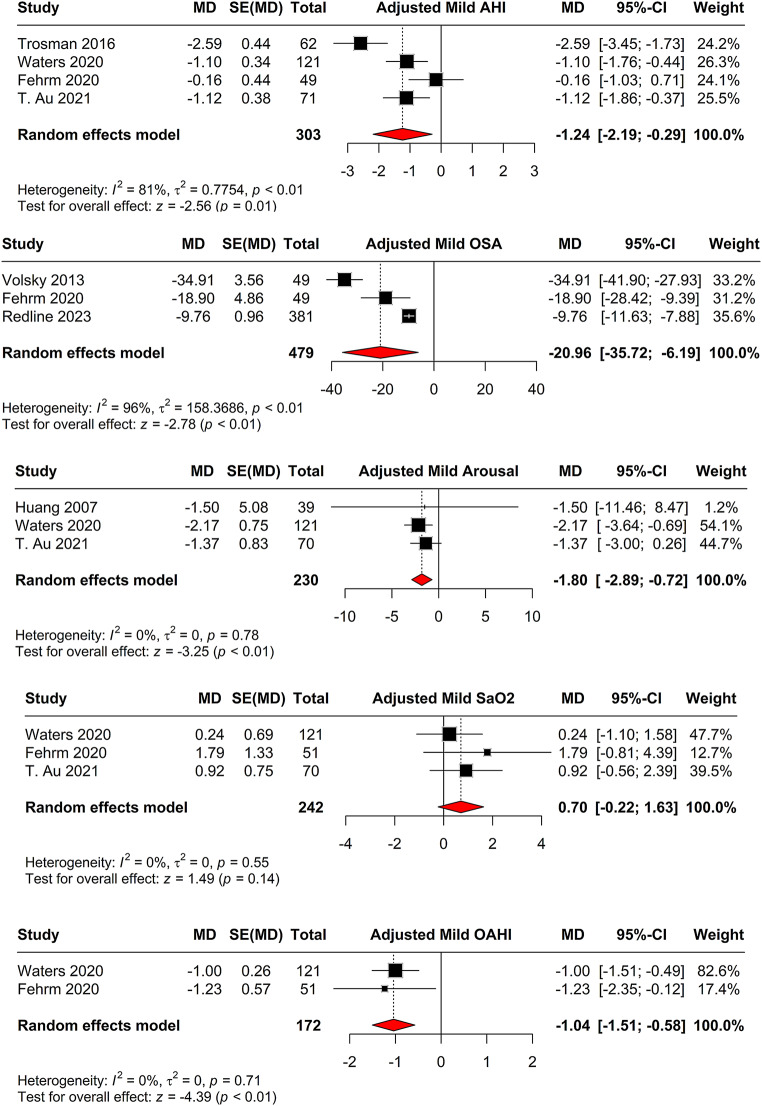
ANCOVA-adjusted comparison of adenotonsillectomy versus watchful waiting. The forest plots show MDs for AHI, arousal index, SaO2, OAHI, and OSA scores after adjusting for baseline values

After conducting the ANCOVA test adjusting for baseline, four studies indicated an MD of -1.24 (95% CI = [-2.19; -0.29], *p* = 0.011) for AHI, with high heterogeneity (I^2 = 80.7%) (Fig. 6). The arousal index, adjusted for baseline in three studies, showed an MD of -1.80 (95% CI = [-2.89; -0.72], *p* = 0.001), with no heterogeneity (I^2 = 0.0%) (Fig. 6). SaO2, adjusted for baseline in three studies, resulted in an MD of 0.70 (95% CI = [-0.22; 1.63], *p* = 0.14), with no heterogeneity (I^2 = 0.0%) (Fig. 6). For OAHI, adjusted for baseline in two studies, the MD was − 1.04 (95% CI = [-1.51; -0.58], *p* < 0.001), with no heterogeneity (I^2 = 0.0%) (Fig. 6). OSA, adjusted for baseline in three studies, indicated an MD of -20.96 (95% CI = [-35.72; -6.19], *p* = 0.005), with high heterogeneity (I^2 = 95.9%) (Fig. 6).

### Reported safety related data among the included studies

AT for children with mild SDB has been found generally safe across the included studies but not without potential complications. Common issues include postoperative desaturation events, particularly during REM sleep, which typically resolve within six weeks [25]. Oxygen desaturation requiring intervention has found to be more common in younger children and those with asthma [26]. Postoperative bleeding occurs in a minority of cases, with varying severity, and some children may experience perioperative respiratory complications [27]. Serious adverse events are rare, with no long-term problems reported [28]. For a detailed breakdown, refer to Table 2. Overall study therefore supports the overall safety of both surgery and watchful waiting but suggests the need for ongoing monitoring of children treated conservatively.

**Table 2:** Reported complications among the included stıudies

Study	Complications
Nixon et al., 2005	“Sleep disturbance-reduced REM sleep, airway obstruction, obstructive apneas, hypopneas (these complications were seen particularly in severe OSA), desaturation. More apnea and desaturation were observed in REM sleep than in quiet sleep, copious nasal secretions, postop edema. Obstructive events were observed in all patients on the first night after surgery. The incidence of obstructive apneas and hypopneas was approximately four times higher in the severe OSA group compared to the mild OSA group, and this was linked to more significant desaturation. Desaturation occurred in all 10 patients included in this study, caused by upper airway obstruction. The desaturation events were primarily caused by obstructive apneas and hypopneas, especially in the severe OSA group. Desaturation had resolved 6 weeks after surgery. There was a tendency for increased apnea and desaturation during REM sleep compared to quiet sleep. Only one patient needed intervention due to respiratory compromise. Twelve hours post-adenotonsillectomy, mask oxygen was initiated after repeated obstructive apneas led to desaturations below 80%. The patient remained in the hospital for three nights until the desaturation episodes during sleep were resolved.”
Gan et al., 2015	“Eleven children (21.6%) experienced oxygen desaturations while asleep that required intervention, all of which improved with simple supplemental oxygen administration. Within this group, nine children were under the age of 4, and the other two had asthma. Regardless of comorbidities, 9 out of 27 (33.3%) children under the age of 4 experienced desaturations. The only children older than 4 years who experienced desaturations were those with additional comorbidities.”
Hsu et al., 2017	“Fifty-six children (9.2%) experienced postoperative bleeding. Regarding the severity of bleeding, 42 (6.9%) had level I, eight (1.3%) had level II, and six (1.0%) had level III bleeding. (level I, self- or parent-reported postoperative bleeding; level II, requiring inpatient admission for postoperative bleeding; and level III, requiring reoperation to control postoperative bleeding). Only one child had primary bleeding postoperatively. The rates of perioperative respiratory complications varied. The rates for SpO2 levels below 90%, 92%, and 95% in the recovery room were 1.6% (10/610), 3.6% (22/610), and 11.6% (71/610), respectively. Additionally, the rates were 5.4% (33/610), 6.7% (41/610), and 14.3% (87/610) based on SpO2 levels below 90%, 92%, and 95% with additional airway management for perioperative respiratory complications. Six children experienced major respiratory complications; three required endotracheal intubation during recovery, one had postoperative bronchospasm, and two developed postoperative pneumonia, necessitating readmission for intravenous antibiotics. The bleeding rate was consistent among children with varying disease severities. The incidence of perioperative respiratory complications, defined as SpO2 below 90%, 92%, and 95% with additional airway management, significantly increased with the severity of OSA. However, children with different OSA severities did not show significant differences in major respiratory complications. Severe OSA increases the risk of perioperative respiratory complications without increasing the risks of major respiratory or bleeding complications.”
Redline et al., 2023	“Among the children who had adenotonsillectomy, 2.2% experienced a serious adverse event related to the surgery (n = 231). Six serious adverse events were due to perioperative complications, such as secondary bleeding that needed treatment. One child who was initially in the watchful waiting group switched to surgery and experienced one of these events. There were no long-term problems noted from the surgery. After the operation, nonserious pain was reported in 11 patients. Five patients had serious bleeding post-operation, and eight patients had nonserious bleeding. Three patients had nonserious dehydration. One patient had aspiration pneumonia. One patient developed hives due to an allergy to the tape used in polysomnography, and only one patient vomited immediately after a blood draw.”

REM: Rapid Eye Movement; OSA: Obstructive Sleep Apnea; SpO2: Peripheral Capillary Oxygen Saturation

### Quality assessment and risk of bias results of the included studies

We utilized validated tools to assess the quality of 27 studies included in our analysis. We used the ROB2 tool for 8 RCTs; six of them were rated as good quality with low risk of bias [28–33], and the other two studies were of some concern given the nature of non blinding in the both [34, 35]. We used the NIH tool for 19 cohort and cross-sectional studies, 16 of them were of good quality [27, 36–50], 3 studies were of fair quality [25, 26, 51]. Further details can be found in the Supplementary material containing the tables of quality assessment (Table S1-S2).

## Discussion

To our knowledge this is the first comprehensive systematic review and meta-analysis to assess the efficacy of AT in treating mild obstructive sleep OSA in children. The analysis of AT efficacy revealed significant improvements across various sleep-related metrics. The PSQ results showed a notable reduction in scores, indicating better sleep quality. Similarly, OSA metrics improved significantly post-surgery, although with considerable variability among studies. AT led to a significant decrease in the AHI and arousal index, with consistent improvements across studies. When compared to watchful waiting, AT consistently outperformed in reducing AHI and arousal index, even when adjusted for baseline values. However, changes in oxygen saturation were not statistically significant. Overall, AT demonstrated clear benefits in managing pediatric sleep-disordered breathing, especially compared to non-surgical management.

AT is the primary surgical intervention for pediatric OSAS, which is primarily caused by enlarged adenoid tonsils. It is commonly performed worldwide and is of importance in improving quality of life and reducing symptoms and complications associated with OSAS, such as behavioral problems, cognitive deficits, and cardiovascular problems. However, the management of mild OSAS in children remains controversial and there is limited data addressing the benefits of surgery in this group of patients. When comparing children with SDB to healthy controls, a recent meta-analysis highlighted a wide range of neurodevelopmental abnormalities [52]. These deficits are believed to result from sleep loss, sleep fragmentation, and intermittent hypoxemia, all of which can adversely affect developing brain circuitry [53]. Current research debates also primarily focus on attention and executive function, as the frontoparietal attentional network is particularly sensitive to sleep disruptions [54].

Our meta-analysis demonstrated that AT significantly improved PSQ scores in children who underwent the surgery compared to those who were managed with watchful waiting. This finding underscores the potential benefits of surgical intervention in mitigating the neurodevelopmental impacts of OSAS by enhancing sleep quality and reducing the associated cognitive and behavioral deficits. The improvement in PSQ scores suggests that adenotonsillectomy can effectively address the underlying sleep disturbances, thereby supporting better overall neurodevelopmental outcomes for affected children. However, it is important to note that the long-term effects of AT on neurological performance and cognition are not yet fully understood. Current limited data indicate that there may be no significant improvements in cognitive function in children with sleep-disordered breathing [29, 55]. Several potential explanations may be attributed for the lack of observed effect on executive function and attention. Firstly, improvements may be difficult to discern given the wide range of factors influencing cognitive function and test performance. Cognitive testing in controlled conditions may not accurately reflect performance in real-world situations where children need to attend to multiple tasks and may be influenced by suboptimal sleep prior to assessment. Additionally, past studies that have identified an association between cognitive impairment and SDB may have been biased. A large cross-sectional study suggested that the association between habitual snoring and cognition was significantly attenuated after accounting for multiple confounders [56].

Therefore, while AT shows promise in improving sleep quality and related behavioral outcomes, its real long-term effect on neurological performance and cognition remains to be elucidated. Further research with more comprehensive and real-world assessments is needed to fully understand the cognitive impacts of AT in children with SDB.

Our meta-analysis showed that AT had significant improvements in OSA-18. The OSA-18 is the most widely used quality-of-life survey for pediatric SDB and has a positive correlation with condition severity. The analysis of OSA-18 showed a significant mean difference from baseline (MD = -27.47, *p* < 0.001), confirming substantial symptomatic relief. These broad improvements in symptom-based measures are consistent with studies of AT conducted across patient populations with varying levels of AHI [3, 32, 57, 58]. This consistency underscores the effectiveness of AT in enhancing the quality of life for children suffering from SDB, regardless of the initial severity of their condition.

In our analysis, AHI improved significantly as well as OAHI (MD = -1.45, *p* < 0.001; MD = -1.84, *p* < 0.001), reflecting reduced apnea and hypopnea events with a pattern consistent with previous studies [29, 32, 58]. Exploratory analyses in the literature indicate that the effect of AT on the AHI is not associated with age, sex, socioeconomic status, or obesity. However, the impact appears more pronounced in children with greater sleepiness and higher AHI levels [28, 29]. Due to the lack of standardized reporting systems and varying regression analysis adjustment methods among the included studies, we were unable to perform a meta-analysis to better understand the variables that could influence the efficacy of AT on the AHI index. To advance our understanding and improve clinical outcomes, it is crucial that future studies adopt more standardized and unified reporting systems. Consistent methodologies and uniform regression analysis adjustment methods will enable more comprehensive meta-analyses, leading to clearer insights into the factors that affect the success of AT in treating sleep-disordered breathing in children. Researchers, clinicians, and stakeholders must collaborate to establish and implement these standardized protocols with longer follow-up periods, ensuring more reliable and comparable results across studies.

The arousal index also significantly improved (MD = -1.62, *p* = 0.007), indicating better sleep continuity. However, although the oxygen saturation nadir (SaO2) improved the null hypothesis couldn’t be rejected (MD = 1.01, *p* = 0.080). This may be due to the nature of the sample in the included studies being mild OSAS, where oxygen saturation may not be severely compromised initially, limiting the improvements observed after surgery. Overall, these results indicate that AT markedly improved QoL and resolved most sleep disturbances in children with OSA.

The pooled analysis of follow-up between studies comparing AT with watchful waiting revealed significant differences in favor of the AT group for the AHI, Arousal index, OAHI, and OSA-18. While the SaO2 didn’t differ significantly between groups. Moreover, the pooled change from baseline, as well as the baseline-adjusted ANCOVA analysis, showed similar results. Although our investigation focused on patients with mild obstructive sleep apnea, our findings are consistent with existing evidence from systematic review and meta-analysis articles on different severities [52, 55].

The findings of this study must be seen considering some possible limitations. The first limitation is that since we followed the PRISMA guidelines we excluded gray literature and unpublished studies, which might have omitted relevant data. Additionally, some studies had a lack of standardized data reporting and did not classify the severity of the included patients as mild, moderate, or severe, these studies had to be excluded, potentially introducing selection bias. Despite these limitations, there are several strengths of this work such as we strictly adhered to the PRISMA guidelines. We also adjusted baseline values using ANCOVA, thus reducing any possible selection bias within studies.

## Conclusion

In conclusion, this systematic review and meta-analysis confirms the effectiveness of AT in treating children with mild OSAS. Our findings demonstrate that AT significantly improves key indices such as PSQ, OSA-18, AHI, arousal index, and OAHI, thus improving sleep quality and reducing symptoms. However, the long-term effects of AT still require additional controlled studies with extended follow-up periods to determine the effectiveness of AT.

## Data Availability

The datasets generated during and/or analyzed during the current study are available from the corresponding author on reasonable request.
